# Elevated Level of Serum Carcinoembryonic Antigen (CEA) and Search for a Malignancy: A Case Report

**DOI:** 10.7759/cureus.648

**Published:** 2016-06-20

**Authors:** FNU Asad-Ur-Rahman, Muhammad W Saif

**Affiliations:** 1 Internal Medicine Residency, Florida Hospital-Orlando; 2 Hematology/Oncology, Tufts Medical Center

**Keywords:** carcinoembryonic antigen, lithium

## Abstract

Carcinoembryonic antigen (CEA) has been shown to be associated with tumor burden in patients with colorectal cancer. However, it is also elevated to a significant degree in a number of other malignant and non-malignant conditions. We report a case of reversible CEA elevation in a patient using lithium for bipolar disorder.

A 58-year-old female with a longstanding smoking history and a past medical history of chronic obstructive pulmonary disease (COPD), bipolar illness, hypothyroidism, and obesity was found to have an elevated CEA level of 11.2 ng/ml (normal level <5 ng/ml) in the workup for postmenopausal bleeding. Her history was not positive for malignancy of colorectum, ovaries, thyroid, or breast.  She underwent a large number of imaging and endoscopic studies to evaluate for colorectal, breast, ovarian, and lung cancer; however, it did not reveal any evidence of malignancy. Upon review of her medications, she reported that she had recently started lithium for her bipolar illness. We followed up her CEA level while her dose of lithium was reduced from 450 to 300 mg per day. Her CEA level decreased from 25 mg/dl to 6.1 mg/dl and remained stable over the course of the next eight months.

Our case is the first case report that identifies lithium as a potential cause of reversible CEA elevation. The underlying mechanism is yet to be elucidated, but it underscores the importance of investigating the medications as part of the workup.

## Introduction

Carcinoembryonic antigen (CEA) is an oncofetal glycoprotein that is normally expressed by mucosal cells. It is overexpressed by a variety of malignancies. Although it is most commonly associated with colorectal cancer, it can also be elevated in other malignancies such as breast, liver, stomach, and pancreas [1]. However, there are a number of benign conditions that may lead to elevations in serum CEA level including cigarette smoking, pancreatitis, biliary obstruction, peptic ulcer disease, and hypothyroidism, but the extent of elevation is substantially less, and it is rare to see an elevation of >10 ng/ml in this context.

The sensitivity and specificity of CEA levels is a topic of debate for the diagnosis, prognosis, and followup for colorectal cancer. In the case of colorectal cancer, it varies with the tumor stage, grade, location, and spread to the liver. Several studies have shown that patients with high preoperative concentrations of CEA have a worse outcome than those with low concentrations of the marker [2]. With a reference value of 5 ng/ml, the sensitivity of CEA was at 37% only for patients with colorectal carcinoma at Dukes B stage, 66.6% for patients at stage C, and 75% for patients at stage D. The specificities of the CEA for the cancers of the colon and rectum were at 76.98% with a reference value of 5 ng/ml and 86% with a reference value of 10 ng/ml [3].  

Since there are a number of non-malignant etiologies of elevated CEA level, its use as a screening tool for cancers is not encouraged. However, sometimes an accidental finding of an elevated CEA level could lead to an extensive workup that not only heightens a patient’s anxiety but can also impact resources to perform unnecessary diagnostic tests. Lithium is a commonly used medication in the treatment of bipolar disease that may lead to a number of renal, musculoskeletal, and cardiac adverse effects. We report the case of a patient with an elevated CEA level who underwent an extensive investigation for underlying malignancy which was later found to be related to lithium prescribed to the patient for bipolar disorder.

## Case presentation

A 58-year-old female with a 50 pack per year smoking history and a past medical history of chronic obstructive pulmonary disease (COPD), obstructive sleep apnea (OSA), diabetes mellitus (DM), bipolar illness, hypothyroidism and obesity (BMI 31.2 kg/m^2^) presented with an incidentally identified elevated CEA level of  11.2 ng/ml (normal level <5 ng/ml). During an evaluation for postmenopausal bleeding, the patient’s gynecologist ordered CA-125, CA 19-9, and CEA. CA-125 level was 17 Units/ml (normal level <35 units/ml), and CA 19-9 was <3 (normal level <37 units/ml), but CEA was elevated at 11.2 ng/ml.

Given the degree of elevation, it was decided to evaluate for underlying malignancy. She did not report any weight loss, chronic diarrhea or constipation, hematochezia, melena, or abdominal pain.  She then had gastrointestinal evaluation including an upper gastrointestinal (GI) endoscopy and colonoscopy which did not reveal any polyps or evidence of inflammatory bowel disease. Subsequent capsule endoscopy was unrevealing, though of limited quality. Positron emission tomography (PET) scan was negative for hypermetabolic lesions. A subsequent MR enteroscopy was within normal limits. Her last mammogram was also within normal limits.  On gynecologic evaluation, pelvic ultrasonography revealed stable ovarian cysts (measuring 3 mm). An endometrial biopsy did not reveal any dysplasia. TSH level was 3.4 units/ml (normal level 0.5 to 4.5 units/ml), and free T4 was 1.10 ng/dl (normal range 0.8 to 2.7 ng/dl).

She was followed-up over eight months, and a decline of the CEA level was noted from 25 mg/ml to 6.1 ng/ml when the dose of lithium was decreased from 450 to 300 mg daily in light of supratherapeutic lithium levels. A subsequent followup was done after three months and the CEA level remained within normal limits. The patient agreed to participate and was explained the nature and objectives of this study, and informed consent was formally obtained. No reference to the patient's identity was made at any stage during data analysis or in the report.

## Discussion

Our case illustrates an extensive workup that was initiated after a CEA level was found to be incidentally elevated greater than 10 ng/ml. Studies have shown that CEA levels greater than 10 ng/ml are rarely secondary to nonmalignant conditions, and the subsequent investigations were based on the aforementioned observation [4]. However, all of the investigations were negative, and the CEA level declined after a decrease in the dosage of lithium that the patient was using for her bipolar disorder.

The causes of an elevated CEA level are listed in Table 1.

**Table 1:** Common causes for an elevated carcinoembryonic antigen (CEA) level

Colorectal Cancer
Primary ovarian cancer
Breast cancer
Thyroid cancer
Non-small cell lung cancer
Cigarette smoking
Mucinous cystadenoma of ovary/appendix
Cholecystitis
Liver cirrhosis
Pancreatitis
Inflammatory bowel disease
Medications like orlistat

Lithium is a widely used in mood disorders. It has been associated with increased risk of nephrogenic diabetes insipidus, hypothyroidism, hyperparathyroidism, and weight gain [5]. Lithium increases intrathyroidal iodine content, inhibits the coupling of iodotyrosine residues to form iodothyronines (thyroxine [T4] and triiodothyronine [T3]), and inhibits the release of T4 and T3 in therapeutic doses [6]. Given the association of hypothyroidism and medullary thyroid cancer with elevated CEA levels, we ordered serum TSH, free T4, and thyroglobulin levels. All these lab results came back within normal limits. This ruled out hypothyroidism or medullary thyroid cancer as a potential cause of the elevated CEA level.

The exact mechanism of an association of lithium and an elevation of CEA level is unknown and would need further studies to elucidate the pathophysiology. To our knowledge and literature search, this is the first case report linking lithium with an elevated CEA level. Ferandes G et al., reported a case of CEA elevation with orlistat, which resolved after discontinuation of orlistat [7]. The underlying pathophysiology by which orlistat led to CEA elevation was unclear. Theoretically, orlistat's inhibition of pancreatic lipase can lead to colitis from unabsorbed lipids which can lead to an elevated CEA level. However, this does not appear to be a similar pathway for lithium. This emphasizes the need to reassess the medications in patients with elevated CEA levels and to consider lithium in the differential diagnoses. As lithium is not an uncommon medication in the outpatient setting, routine measurement of CEA levels should be obtained in patients on this medication to further confirm this association.

## Conclusions

In conclusion, the use of CEA testing has been shown to be of prognostic significance in patients with colorectal cancer, especially with hepatic metastasis. Its nonspecific nature in other settings can lead to a significant workup that may not ultimately affect the management of patients. We report the first case report incriminating lithium as an underlying reason for CEA elevation. Physicians should be cautious about ordering such tests unless clinically indicated, and if found abnormal, non-malignant causes--especially medications--must be reviewed.
